# IL-1RAP, a Key Therapeutic Target in Cancer

**DOI:** 10.3390/ijms232314918

**Published:** 2022-11-29

**Authors:** Jame Frenay, Pierre-Simon Bellaye, Alexandra Oudot, Alex Helbling, Camille Petitot, Christophe Ferrand, Bertrand Collin, Alexandre M. M. Dias

**Affiliations:** 1 Plateforme d’Imagerie et Radiothérapie Précliniques, Médecine Nucléaire, Centre Georges-François Leclerc, 21000 Dijon, France; 2 INSERM UMR1098, EFS BFC, Université de Bourgogne Franche-Comté, 25000 Besançon, France; 3 CanCell Therapeutics, 25000 Besançon, France; 4 Institut de Chimie Moléculaire de l’Université de Bourgogne, UMR CNRS 6302, 21000 Dijon, France

**Keywords:** cancer, IL-1RAP, IL-1R family, innovative therapies, metastasis

## Abstract

Cancer is a major cause of death worldwide and especially in high- and upper-middle-income countries. Despite recent progress in cancer therapies, such as chimeric antigen receptor T (CAR-T) cells or antibody-drug conjugate (ADC), new targets expressed by the tumor cells need to be identified in order to selectively drive these innovative therapies to tumors. In this context, IL-1RAP recently showed great potential to become one of these new targets for cancer therapy. IL-1RAP is highly involved in the inflammation process through the interleukins 1, 33, and 36 (IL-1, IL-33, IL-36) signaling pathways. Inflammation is now recognized as a hallmark of carcinogenesis, suggesting that IL-1RAP could play a role in cancer development and progression. Furthermore, IL-1RAP was found overexpressed on tumor cells from several hematological and solid cancers, thus confirming its potential involvement in carcinogenesis. This review will first describe the structure and genetics of IL-1RAP as well as its role in tumor development. Finally, a focus will be made on the therapies based on IL-1RAP targeting, which are now under preclinical or clinical development.

## 1. Introduction

While cardiovascular diseases remained the major cause of death worldwide, a recent report highlights cancer as the leading cause of death in high-income countries and in some upper-middle-income countries showing a transition in the predominant causes of death in the near future [1]. The high level of heterogeneity characterizing cancers is the major reason for therapeutic failure in patients [2,3]. The development of chemotherapy was a major revolution in the care of cancer patients decades ago [4,5,6]. Despite the global improvement in cancer outcomes brought about by chemotherapy, the lack of specificity and the disabling associated side effects of systemic treatments induce a high heterogeneity of responses among patients and cancer types, as well as the emergence of resistance, which are major clinical concerns [7,8]. In order to improve the impact on patient outcomes, the new therapies are more personalized and better target antigens that are specific for cancer cells. Yet, even if these new approaches show better results than usual chemotherapies, every new treatment tends to narrow the pool of patients that could benefit from this strategy.

To broaden the pool of treatable patients without losing the specificity of targeted therapies toward tumor antigens, other approaches emerged. One of them is to target ubiquitous inflammatory/pro-tumoral proteins which are abnormally upregulated by tumor cells [9]. It is well known that both inflammation and tumorigenic processes have intricate relationships [10,11,12]. 

Acute inflammation is a desirable response to tissue damage or infection initiated by dendritic cells or macrophages secreting pro-inflammatory mediators (such as cytokines). The inflammatory response will induce the dilation of blood vessels and the increase in their permeability, thus enabling the recruitment of leucocytes and plasma proteins (e.g., albumin and fibrinogen) to restore tissue damage or fight infection. Under normal conditions, once the offending agent is eliminated, the inflammatory process ends with the termination of the inflammatory cascade and regain of homeostasis [13]. Yet, the inflammatory process can be impaired at different stages due to several stimuli (i.e., genetic mutations in senescent cells, bacterial or viral infection, inflammatory diseases and environmental-related stimuli), resulting in chronic inflammatory conditions thought to be associated with cancer development and progression [14,15]. This cancer-related inflammation was divided into two pathways: (i) an intrinsic pathway when genetic events are causing inflammation and neoplastic transformation, (ii) an extrinsic pathway when inflammation is caused by infections or environmental exposure and can promote carcinogenesis [16]. Both pathways will lead to a final state of chronic inflammation which will have a prominent role in the initiation, promotion, and proliferation of cancer cells.

In this context, numerous cytokines and their pathways are broadly over-expressed among the different kinds of cancer, representing potential therapeutic targets [10,17,18]. Among all proteins that showed a significant pro-tumoral effect, the interleukin-1 receptor accessory protein (IL-1RAP) appears as a valuable target for several kinds of cancers [19]. IL-1RAP is involved in three signaling pathways of the IL-1 family: interleukin-1 receptor (IL-1R), interleukin-33 receptor (IL-33R) and interleukin-36 receptor (IL-36R) [20]. These pathways display pro-tumoral activities [21], and IL-1RAP was found upregulated in several cancers, including hematological tumors (i.e., myeloblastic and lymphoblastic leukemias) [22,23] as well as solid tumors (i.e., pancreatic, colorectal or breast neoplasms) [24,25,26].

As this protein shows great therapeutic potential, two therapies that target IL-1RAP are currently undergoing clinical trials with one chimeric antigen receptor T-cells (CAR-T) therapy [27] and two immunotherapies using an antibody to activate the antibody-dependent cell-mediated cytotoxicity (ADCC) or by direct blockade of IL-1RAP [24,28].

After a quick overview of the relationships between cancer and inflammation, this review will describe the IL-1 family with a particular focus on IL-1RAP. Then, the role of IL1-RAP in cancer progression and metastasis formation will be presented. Finally, a focus will be made on the different therapeutic approaches targeting IL-1RAP that went under evaluation.

## 2. Description of the IL-1 Axis & Focus on IL-1RAP

### 2.1. The IL-1 Superfamily

**Figure 1:**
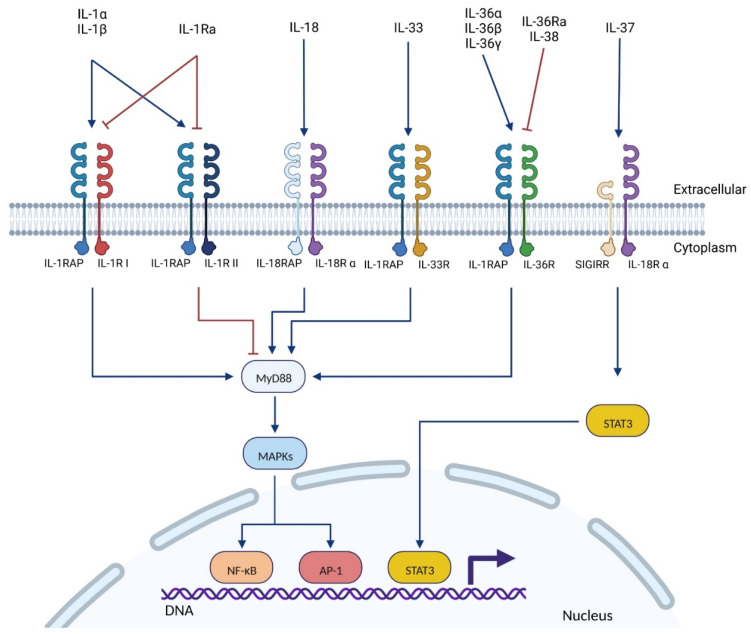
The interleukin 1 superfamily: from the cytokines and their receptors to their main signaling pathway. The ligand (interleukin) binds to its main receptor, which induces the recruitment of the co-receptor (IL-1RAP, IL-18RAP, or SIGIRR). The TIR intra-cellular domains of the receptor/co-receptor complex will recruit MyD88, which activates the MAPK signaling pathway. Finally, MAPKs induce NF-κB or AP-1 transcription factors. The IL-37 is the only interleukin known to activate another pathway, independently of MyD88, which induces the STAT3 transcription factor. Abbreviations: AP-1: activator protein 1; IL: interleukin; IL-1R: interleukin 1 receptor; IL-1Ra: interleukin 1 receptor antagonist; IL-1RAP: interleukin 1 receptor accessory protein; MAPK: mitogen-activated protein kinase; MyD88: myeloid differentiation primary response 88; NF-κB: nuclear factor kappa-light-chain-enhancer of activated B cells; SIGIRR: single Ig IL-1-related receptor; STAT3: signal transducer and activator of transcription 3. Created with BioRender.com.

The IL-1 superfamily is composed of numerous cytokines (Table 1) and their receptors (Table 2). Currently, eleven cytokines, five receptors, and five co-receptors have been described among the IL-1 superfamily. While most of them are well described, the exact function of some cytokines (IL-37, IL-38) and receptors (SIGIRR, IL-1RAPL1/2) is not yet fully understood. Members of the IL-1 family are usually split into four subfamilies depending on their co-receptors: (i) IL-1, (ii) IL-33, (iii) IL-36 subfamilies that work with the co-receptor IL-1RAP, and (iv) the IL-18 subfamily that binds a distinct co-receptor (IL-18RAP, Figure 1) [20].

Members of the IL-1 family are known to be key signaling molecules in the innate and adaptive immune systems with a particular role in promoting inflammatory responses. Despite differences between each member of this superfamily, their structure and the mechanism of their signaling pathway initiation are highly conserved [21].

### 2.2. IL-1RAP: The Pro-Inflammatory Co-Receptor of the IL-1 Superfamily

IL-1RAP is a member of the IL-1 receptor family. It was first characterized in 1995 by Greenfeder et al. as a co-receptor of the types I and II IL-1 receptors (IL-1RI and IL-1RII) [54]. IL-1RAP was then further characterized as a co-receptor of the IL-33R [55] and the IL-36R [50] (Figure 1).

It must be noted that three other co-receptors are described in the IL-1 family, initially named after IL-1RAP: IL-18-RAP (also called IL-1RAcPL or IL-1R7) [56], IL-1RAPL1 [52] and IL-1RAPL2 [53]. Despite some similarities in the biological activities of those proteins compared to IL-1RAP, this review will not further describe their characteristics.

#### 2.2.1. Genetics and Structure of IL-1RAP

In 1997, Dale et al. discovered that the gene of IL-1RAP is encoded in chromosome 3q28 and independently from the IL-1 receptor family cluster encoded on chromosome 2q12 [57].

Radons et al. [58] studied, in 2003, the effects on the signal transduction of several mutations in the TIR (toll/Interleukin-1 receptor) domain of IL-1RAP. They generated mutants that were tested in vitro to determine which amino acids were essential to the IL-1 signal transduction. Their results showed two critical regions in the TIR domain of IL-1RAP: (1) the Pro-446, whose substitution by a charged amino acid such as Histidine impaired the interaction of IL-1RAP with other TIR domains and MyD88, both needed for IL-1 signaling; (2) the amino acids 527 and 534 which are both needed to keep an effective 3D structure of IL-1RAP. Moreover, the deletion of both amino acids 527 and 534 is necessary to impair IL-1 signaling.

**Figure 2:**
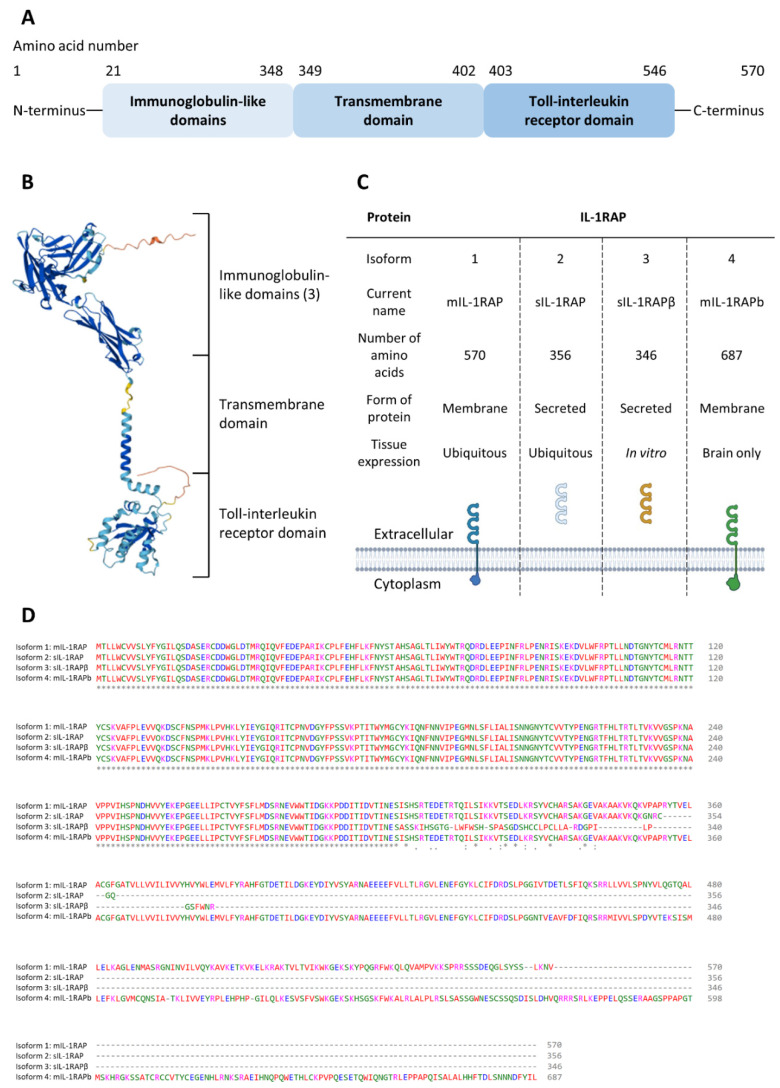
Structure of IL-1RAP and homogeneity between its different isoforms. (**A**): Description of the different domains of the mIL-1RAP structure; (**B**): Predicted 3D structure of IL-1RAP (AlphaFold ID: AF-Q9NPH3-F1); (**C**): Characteristics of the four isoforms of IL-1RAP; (**D**): Protein alignment (Clustal Omega) between the four isoforms of IL-1RAP.

There is a great homology within the molecular structures of all proteins of the IL-1R superfamily. Like most of the other receptors from this family, the molecular structure of IL-1RAP is composed of a triple-immunoglobulin (Ig) ectodomain (sometimes called D1, D2, and D3), which can recognize β-trefoil class cytokines and a TIR C-terminal endodomain which is able to activate NF-κB and MAPK pathways [59] (Figure 2A,B).

The 3D structure of the ectodomain of IL-1RAP has already been described as a complex with the associated cytokine and primary receptor. For instance, Casadio et al. characterized the complex of IL-1RAP with IL-1 and IL-1-R I/II in 2001 [60]. Lingel et al. showed the interaction of IL-1RAP with IL-33 and ST2 in 2009 [59], and Liu et al. further characterized the latter in 2013 [38]. Finally, Yi et al. described the complex of IL-1RAP with IL-36 and IL-1-Rrp2 in 2016 [61].

In spite of great sequence divergence, the interaction mechanism between cytokines and their related receptor tends to be strongly conserved within the IL-1 family [62]. This was confirmed by the characterization of the binding of IL-1, IL-33 and IL-36 with IL-1RAP [59,60,61], as in each case, their interaction with IL-1RAP was identical to their interaction with other IL-1 receptors and the formed complexes induced the same signaling pathways (MyD88, MAPK and NF-κB).

It has been shown that a small hydrophobic patch is present in the ectodomain of IL-1RAP, which is needed to bind IL-1Rs. This patch is formed by three amino acid residues: Ile 135, Leu 180, and Ile 181 [38]. The involvement of this region in the binding with the other receptors IL-33 and IL-36 has not been fully investigated yet. Furthermore, during the recruitment of IL-1RAP by IL-36R, it has been observed that the D2 and D3 extracellular domains of IL-1RAP interact with the D2 and D3 extracellular domains of the primary receptor, respectively [61]. Even if this interaction was only characterized with IL-36R for now, it is likely that similar interactions with the other receptors of the IL-1 superfamily occur. Indeed, it has been shown that all the receptors from this family present a great conservation in their extracellular domains of cysteine residues [61], which could form disulfide bonds and thus promotes the 3D structure of the receptors. 

Liu et al. also described the rigidity of IL-1RAP in the structure with IL-33 and ST2 [38]. They suggested that the structural rigidity of IL-1RAP explains its inability to bind the cytokine in a direct manner. Indeed, the ectodomain of the primary receptor is more flexible, thus justifying that it is able to bind its ligand. Furthermore, they supposed that their model for the interaction between IL-1RAP and IL-33–ST2 could be similar to the other members of the IL-1 family.

As with many other proteins, IL-1RAP can be expressed in several isoforms through alternative splicing. The literature describes 4 isoforms for IL-1RAP: 2 soluble forms and 2 membrane forms (Figure 2C).

1. mIL-1RAP: the first form that was described in 1995 by Greenfeder et al. [54]. This mIL-1RAP was first called IL-1-R3, yet IL-1RAP (or IL-1RAcP) is now the usual designation. The mIL-1RAP is involved in the immunity process, and it was recently described in synaptogenesis as being involved in synaptic differentiation [63];
2. sIL-1RAP: This isoform is excreted by the cells and is formed by the extracellular domain of mIL-1RAP. The action of sIL-1RAP was explored by Smith et al. in 2003 [64]. They showed that sIL-1RAP could bind the soluble form of IL-1RII and increase its affinity for both IL-1α and IL-1β. However, sIL-1RAP did not increase the affinity of sIL-1R-II for the receptor’s antagonist, IL-1ra. Thus, they suggested that sIL-1RAP plays a role in the negative regulation of IL-1 signaling;
3. sIL-1RAP β: in 2003, Jensen and Whitehead discovered a second soluble isoform when cells were treated with staurosporine, which is an apoptosis inducer and an inhibitor of protein kinases. They suggested that under stress conditions, the splicing machinery was shifted to produce sIL-1RAP β instead of mIL-1RAP. Thus, the increase of both the soluble isoforms and the decrease of the membrane form led to the inhibition of IL-1 signaling in order to induce the apoptosis mechanism of the cells. Yet, it appeared that other apoptotic inducers, such as UV light, did not lead to the production of sIL-1RAP β [65];
4. IL-1RAPb (also called AcPb): IL-1RAPb structure differs from mIL-1RAP by an extended C-terminal domain (addition of 140 amino acids) and an altered TIR domain;
5. This membrane isoform is typically found in the brain and shows different ways of action than classic mIL-1RAP. Indeed, it was first demonstrated in 2009 [66] that IL-1RAPb did not bind the classical IL-1 family signaling pathways protein (MyD88, IRAK-4) and so did not activate the MAPK signaling. Then, in 2011 [67], IL-1RAPb was described as having a role in modulating excitatory neurotransmission by regulating the activation of the kinase Src and NMDA function.

Using the Clustal Omega [68] software to study the protein alignment of the four isoforms of IL-1RAP, we observed that despite the alternative splicing process, the four isoforms show a 100% homology in their Ig-like domains (Figure 2D). Thus, this demonstrates the importance of this region of the protein.

#### 2.2.2. Location & Signaling Pathways

IL-1RAP is a ubiquitous protein. Yet, its expression level varies among tissues: according to several data banks (such as NCBI, Uniprot, and Bgee), IL-1RAP is mostly expressed in the liver, the placenta, and white blood cells (NCBI Gene ID 3556; UniProtKB ID Q9NPH3; Bgee Gene ID ENSG00000196083). 

IL-1 receptors are found in two main forms, IL-1RI and IL-1RII. IL-1RI is mainly found in T cells and fibroblasts, while IL-1RII is predominantly found in B cells and neutrophils. It appears that IL-1RAP cannot directly bind IL-1 but forms a complex with IL-1Rs, which binds IL-1 with higher affinity than the IL-1Rs alone (IL-1R I NCBI Gene ID 3554; IL-1R II NCBI Gene ID 7850) [54]. 

The IL-33 receptor, ST2, can be expressed in four isoforms, but two of them are predominant: the transmembrane form ST2L, which is constitutively expressed in hematopoietic cells; the soluble form sST2, which is largely inducible and almost ubiquitous [69] (NCBI Gene ID 9173).

The IL-36 receptor, IL-1Rrp2, is mainly found in skin tissues [70], the intestinal *lamina propria* [71], and T and B cells (NCBI Gene ID 8808).

**Figure 3:**
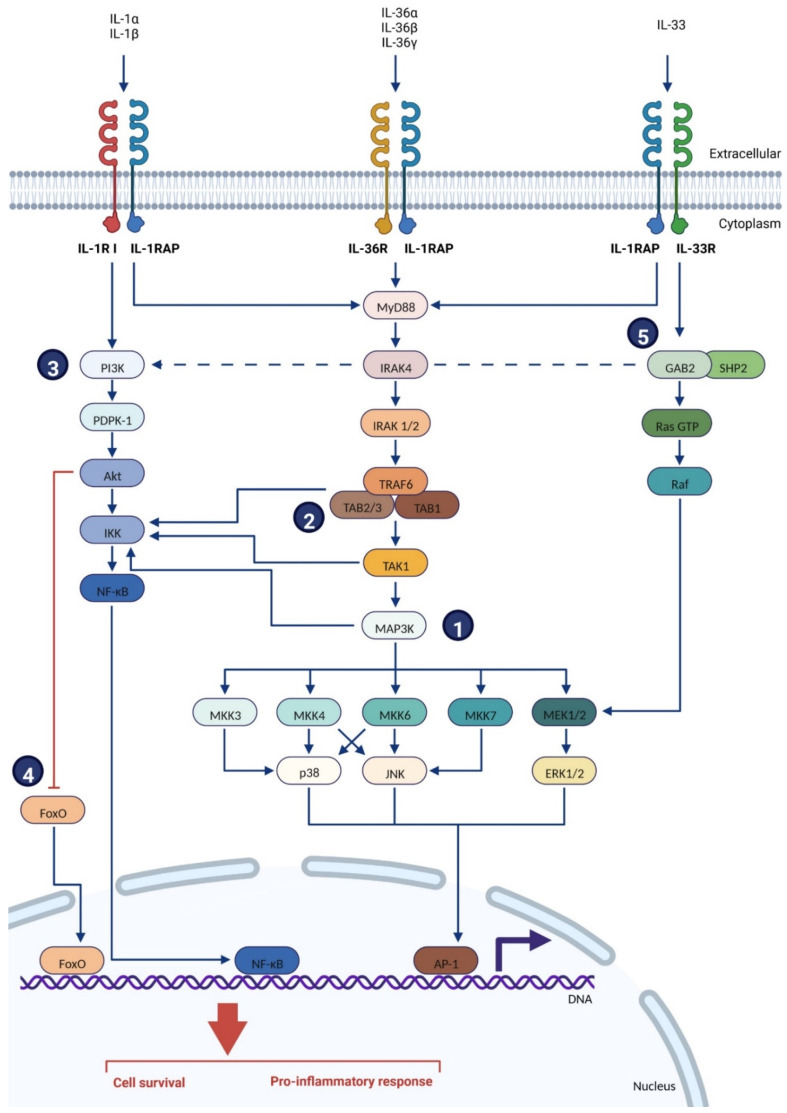
Intracellular signaling pathway of IL-1RAP with IL-1R I, IL-33R or IL-36R [20,31,72,73,74,75,76,77,78,79,80,81,82,83,84,85,86,87,88,89,90]. The interleukin binds to its main receptor, which induces the recruitment of the co-receptor IL-1RAP. The TIR intra-cellular domains of the receptor/co-receptor complex will recruit MyD88, which activates IRAK4 and IRAK1/2. This complex will then trigger TRAF6, which induces a kinases cascade through MAP3Ks, activating MAPKKs (1) and then stimulating the MAPKs p38, JNK, or ERK1/2. This will finally induce the AP-1 transcription factor. (2) TRAF 6 can also activate IKK, which induces the NF-κB transcription factor. (3) NF-κB can also be stimulated independently from TRAF 6 by IL-1R I and IL-33R (thanks to GAB2) through the activations cascade of PI3K, PDK-1, Akt and IKK. (4) Akt activation will also induce the inhibition of FoxO. (5) IL-33R is also able to stimulate AP-1 independently from TRAF 6 by the activations cascade of GAB2, and Ras/Raf signaling, which activates MEK1/2. **Abbreviations:** Akt: protein kinase B; AP-1: activator protein 1; ERK: extracellular signal-regulated kinase; FoxO: forkhead box proteins O; Gab2: GRB-associated-binding protein 2; IKK: IkappaB kinase; IL: interleukin; IL-1R I: interleukin 1 receptor type 1; IL-1RAP: interleukin 1 receptor accessory protein; IRAK: interleukin-1 receptor-associated kinase; JNK: c-Jun N-terminal kinase; MAPK: mitogen-activated protein kinase; MAP3K: mitogen-activated protein kinase kinase kinase; MKK: mitogen-activated protein kinase kinase; MyD88: myeloid differentiation response 88; NF-κB: nuclear factor kappa-light-chain-enhancer of activated B cells; NIK: NF-κB-inducing kinase; p38: MAPK p38; PDPK1: phosphoinositide-dependent Kinase-1; PI3K: phosphoinositide 3-kinase; Raf: “rapidly accelerated fibrosarcoma;” Ras GTP: “rat sarcoma virus” guanosine triphosphatase; SHP2: Src homology region 2 domain-containing phosphatase-2; TAB: TGF-beta activated kinase 1 (MAP3K7) binding protein; TAK1: mitogen-activated protein kinase kinase kinase 7 (MAP3K7); TRAF: TNF receptor-associated factors. Created with BioRender.com.

A general model for the binding of the IL-1 family cytokines (IL-1α, IL-1β, IL-33, IL-36α, IL-36β and IL-36γ) with their primary receptor (IL-1R I and IL-1R II, IL-33R/ST2, Il-36R/IL-1Rrp2) and IL-1RAP was described as follow (Figure 3): the cytokine binds its primary receptor, inducing the recruitment of IL-1RAP. The intracellular TIR (Toll/Interleukin-1 receptor) domains of both the primary receptor and IL-1RAP are juxtaposed, which is necessary to recruit and bind several intracellular proteins and kinases, such as Toll-interacting protein (Tollip), myeloid differentiation factor 88 (MyD88), members of the IL-1R associated kinase (IRAK) family, and TNF receptor-associated factor 6 (TRAF-6). These proteins then trigger intracellular signaling cascades that induce the NF-κB and AP-1-dependent expression of pro-inflammatory cytokines, chemokines, and secondary mediators of the inflammatory response [31] (Figure 3).

## 3. Role of IL-1RAP in Tumors

### 3.1. Expression of IL-1RAP in Cancer

**Figure 4:**
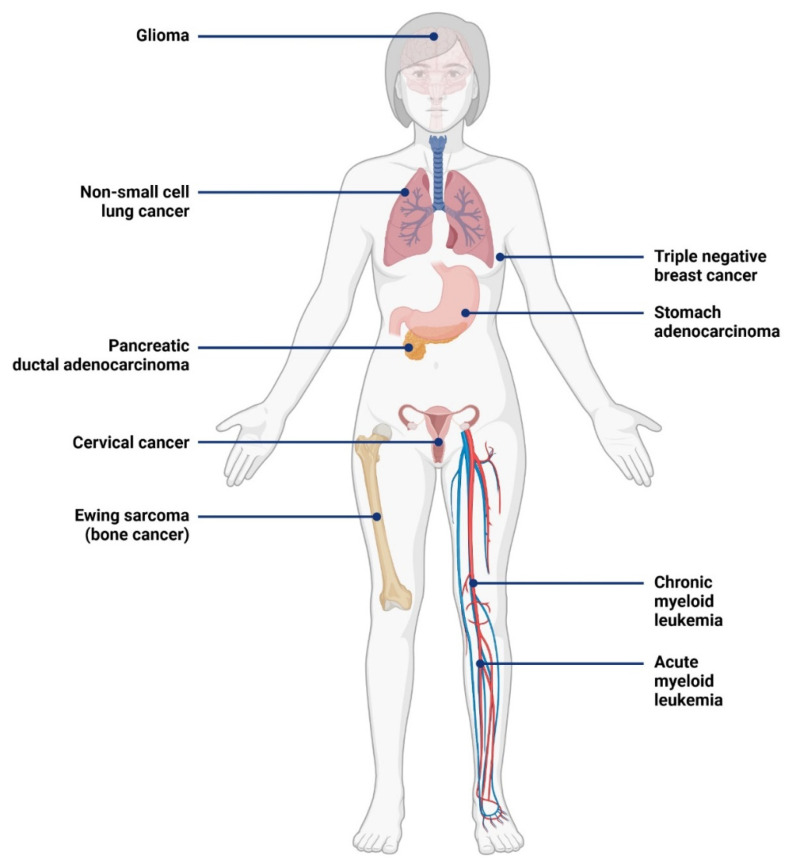
Cancer types with demonstrated IL-1RAP overexpression [24,25,28,93,95,101,106,110,112]. Created with BioRender.com.

As described previously, IL-1RAP is highly involved in the pro-inflammatory response through NF-κB and AP-1 pathways. Because of the involvement of the inflammation process in the development of cancer, the expression of IL-1RAP within the tumor microenvironment and at the surface of the tumor cells was widely explored during the last decades. Today, an overexpression of IL-1RAP has been described in nine types of cancer, as shown in Figure 4.

Firstly, IL-1RAP overexpression was observed in hematological cancers, such as chronic myeloblastic leukemia (CML) and acute myeloblastic leukemia (AML).

CML is associated with a recurrent genetic aberration, the Philadelphia (Ph) chromosome, formed through the translocation between chromosomes 9 and 22 and giving the constitutively active tyrosine kinase P210 BCR/ABL1 [91,92]. Järås et al. demonstrated that the expression of IL-1RAP was strongly correlated with the presence of the Philadelphia chromosome in primitive CML cells. Indeed, they showed that CML Ph+ cells presented an up-regulation of IL-1RAP expression, whereas almost all CML Ph- cells did not present any up-regulation. Thus, IL-1RAP could be used in CML to precisely target CML cells with the BCR/ABL1 mutation [93].

AML is characterized by immature hematopoietic progenitor cells (called “blasts”) that lost their ability to differentiate. Thus, these cells continuously grow and divide without maturation and function and tend to suppress normal hematopoiesis. Immature blasts reside in the bone marrow, peripheral blood, and extramedullary tissues. AML presents several heterogeneous genetic mutations, and more than 60 genes are described as being recurrently mutated in AML. Some of them are frequently gained at relapse and are associated with poor outcomes, such as *DNMT3A*, *ASXL1*, *RUNX1*, and internal tandem duplications in *FLT3* (*FLT3*-ITD) [94]. In accordance with previous results on the expression of IL-1RAP in AML [95,96], IL-1RAP was recently reported to be overexpressed in the leukemic stem cells from the bone marrow of 65% of AML patients [97].

Secondly, IL-1RAP was also described as overexpressed in several solid cancers, affecting different organs.

Pancreatic ductal adenocarcinoma (PDAC) is the most common cancer of the pancreas and is highly aggressive. It presents tubular adenocarcinoma of the ductal glands and is usually surrounded by multiple blood and lymphatic vessels. Thus, the spreading of the tumor cells is greatly facilitated. PDAC presents a poor prognosis due to limited therapeutic options [98]. Using immunohistochemistry (IHC) and single-cell RNA-seq, IL-1RAP was found overexpressed in PDAC patient samples (81%). IL-1RAP was also observed overexpressed in 14 PDAC cell lines using flow cytometry, with the highest expression on human A6L cell lines [25,99].

IL-1 RAP has also been studied in Ewing sarcoma, the second most frequent bone tumor of childhood and adolescence, which is characterized by small round cells overexpressing CD99. Metastases are present in 20–25% of cases and are often resistant to intensive therapy. Ewing sarcoma is characterized by a tumor-specific chimeric transcription factor, EWSR1-FLI1, which massively rewires the transcriptome [100]. High levels of IL-1RAP mRNA were observed in databanks, and the protein overexpression was confirmed by IHC both on patient samples and xenograft of TC32 cells in male NSG mice. The high protein expression levels were also confirmed by Western blotting across multiple Ewing sarcoma cell lines [101].

Non-small cell lung cancer (NSCLC) is a heterogeneous class of tumors that represents 85% of lung cancers. The main risk factors associated with NSCLC are tobacco smoking, air pollution and radon exposure. Yet, oncogenic mutations were also found to be implicated in driving NSCLC. These oncogenes include activating mutations in the epidermal growth factor receptor (EGFR) gene and translocations of the anaplastic lymphoma kinase (ALK) gene [102]. IL-1RAP was found overexpressed in NSCLC cell lines [103], and anti-IL-1RAP immunotherapy was recently successfully assessed in an NSCLC PDX preclinical [28] model, LU2503, on Balb/c mice.

Gliomas represent about 80% of malignant brain tumors that affect the central nervous system and the spinal cord. Glioma cells derive from normal glial cells (astrocytes, oligodendrocytes and ependymal cells), but this process is not fully unraveled yet [104,105]. IL-1RAP was found overexpressed in vitro by western blotting in two glioma cell lines, M059 J and U373 [106].

Triple-negative breast cancer (TNBC) comprises 10–15% of all breast cancers and is characterized by the lack of estrogen receptor (ER), progesterone receptor (PR), and human epidermal growth factor receptor (HER-2). TNBC shows a poor prognosis with high aggressiveness and metastatic capacity. Because the usual endocrine therapy and anti-HER2 immunotherapy used in other subtypes of breast cancers have no effect on these tumors, they remain the hardest subtype of breast cancer to treat [107,108]. IL-1RAP expression was assessed in 30 primary breast cancer tissues by RT-qPCR, depending on their hormone receptors (HR) and HER-2 status. The highest IL-1RAP expression was found in TNBC patients (HR^-^/HER-2^-^). IL-1RAP was also found overexpressed in the two TNBC cell lines, MDA-MB-231 and HCC-70, by RT-qPCR and western blotting [24].

In stomach adenocarcinoma, the *Helicobacter pylori* (Hp) infection was initially considered one of the main factors in its development due to the chronic inflammatory state that the immune system will induce in the gastric mucosa. Yet, it seems that Hp infection is not carcinogenic *per se* and needs other factors to induce carcinoma. A high level of gastrin or long-term treatment with proton pump inhibitors seems to be the upstream cause that leads to stomach carcinoma [109]. Because of the role of chronic inflammation in stomach adenocarcinoma, IL-1RAP protein and mRNA expression was assessed in tissue samples from 25 patients diagnosed with stomach carcinoma, using western blotting and RT-PCR, respectively. The same was done in cell lines (GES-1, AGS, SGC-7901, MGC803). IL-1RAP was found overexpressed in patient samples and in the MGC803 cell line [110].

Cervical cancer is mostly caused by human papilloma virus (HPV) infection, and 99,7% of patients with cervical cancer also present HPV infection. 12 HPV types are described as carcinogenic, with HPV-16 (50%) and HPV-18 (10%) as the most common [111]. In silico analyses of GEO and Oncomine data revealed that IL-1RAP expression was increased in cervical cancer tissues compared to normal tissues. The IL-1RAP expression level was then analyzed in tissue samples from 10 patients with cervical cancer using RT-PCR. IL-1RAP expression was found to increase in all tumor samples of patients compared to their adjacent non-cancerous tissues [112].

Finally, it can be noted that IL-1RAP is also involved in other pathologies, such as inflammatory diseases [113]. IL-1RAP was also described as a potential biomarker (i) in obesity by analyzing plasma levels of its soluble form sIL-1RAP [114], and (ii) in acute anterior uveitis where IL-1RAP gene could be a genetic risk maker in the Chinese population [115].

### 3.2. Role of IL-1RAP in Tumor Progression and Metastases

IL-1RAP is overexpressed in several solid and hematological cancers. Yet, the development of a tumor is characterized by multiple phases during which genetic mutations will appear: initiation, promotion, malignant conversion, and progression. During this tumor development, metastasis [116] may emerge, depending on the cancer type and aggressiveness [117,118,119].

During the initiation stage, epigenetic changes will lead to the activation of protooncogenes of the inactivation of tumor-suppressor genes, which is an irreversible process. Genetic mutations will start to accumulate in the so-called “initiated cells.”

The promotion stage comprises the selective clonal expansion of the initiated cells. The more the cells will divide, the more genetic mutations will appear, thus increasing the risk of gaining a mutation leading to malignant conversion. During this phase, the “preneoplastic cells” also acquire resistance to cytotoxicity (or immune escape capacity) and defects in apoptosis.

The malignant conversion is characterized by preneoplastic cells that now present malignant phenotypes after further genetic mutations. The cells will grow and divide faster than the other tumorigenic cells (the initiated and preneoplastic cells), rapidly becoming the main tumor cells that compose the tumor mass.

During the progression phase, more genetic mutations on protooncogenes or tumor suppressor genes will be gained by the cells, resulting in more aggressive phenotypes over time. Some cancer cells can also acquire the ability to spread to other organs by invading the lymphatic and blood systems and are then defined as metastasis [117].

Metastasis formation is a complex process that can start at the early or late stage of tumorigenesis and which comprises multiple sub-steps: (i) invasion, when tumor cells crosstalk with stromal cells to start moving through the extracellular matrix (ECM); (ii) intravasation, when the tumor cells go from the ECM into the blood vessels; (iii) survival maintenance, when the tumor cells must rewire their metabolism to resist *anoikis*–a mechanism resulting in cell death due to the loss of attachment to the extracellular matrix (ECM)–and to avoid immune cells; (iv) extravasation, when the tumor cells leave the blood vessels to migrate in a second organ where they can either enter dormancy or start proliferation; and (v) colonization, when the tumor cells proliferate until becoming a macrometastatic tumor [120].

Even if IL-1RAP was not yet described as being involved in all these phases, it has been shown to play an important role in many of them, i.e., the promotion, the progression, and the metastasis process.

In the beginning, the cell-intrinsic role of IL-1RAP was mostly studied in leukemia models. First, it was observed in vitro that the downregulation of IL-1RAP using shRNAs on human primary AML cells from patients led to a reduced capacity of colony-forming for the primary AML cells and an increase in apoptosis. Second, the use of an antibody targeting IL-1RAP to block its function also showed an increase in apoptosis and differentiation of THP-1 cells (AML cells), alongside a reduction in cell growth. Furthermore, it was noticed in vitro that the anti-IL-1RAP antibody did not induce the apoptosis of healthy cord blood mononuclear cells. Thirdly, the relevance of IL-1RAP expression in AML pathogenesis was addressed in vivo. To achieve that, hematopoietic and progenitor stem cells (HSPCs) from the bone marrow (BM) of knockout (*Il1rap^-/-^*) mice were transduced with the MLL-AF9 fusion oncogene to get a genetic model of AML. The same was done with wild-type (*wt*) mice. These *wt* or *Il1rap^-/-^* BM-HSPCs were then transplanted in *wt* CD45.1 mice. The *Il1rap-/-* group presented a delayed leukemic progression with a better survival rate than the *wt* group [95]. Altogether, these results showed that IL-1RAP plays an important role in AML pathogenicity. Finally, another important result from this study is that activating FLT3–a receptor tyrosine kinase with a significant role in AML–will tend to cancel the benefits of anti-IL-1RAP treatments. This was demonstrated both in vitro and in vivo during the experiments described above when some samples or subjects did not respond to IL-1RAP blocking or knock-down.

Then, the role of IL-1RAP in tumor development was also explored in pancreatic ductal adenocarcinoma (PDAC) [25] and stomach adenocarcinoma [110]. In PDAC, the knock-down of IL-1RAP was achieved in vitro using siRNA on A6L cells. In stomach adenocarcinoma, the knock-down of IL-1RAP was achieved for both in vitro and in vivo experiments by using an active lentivirus (sh-IL1RAP) on MGC803 cell lines. In the PDAC cell line A6L, the inhibition of IL-1RAP reduced the viability of PDAC cells and their colony growth capacity. It also led to decreased invasiveness of PDAC and to a significant G0/G1 cell cycle arrest. Finally, levels of proliferative phospho/activated ERK, MAPK involved in the IL-1RAP pathway, were reduced in cells under IL-1RAP knock-down. In stomach adenocarcinoma, the inhibition of IL-1RAP resulted both in vitro and in vivo in reduced tumor proliferation, migration and invasion. Similarly to previous results, IL-1RAP knock-down also increased apoptosis in vitro in stomach adenocarcinoma. And during in vivo experiments on BALB/c mice, the xenograft tumors presented a lower volume and weight in the IL-1RAP knock-down group, as well as a lower expression of stomach carcinoma biomarker (i.e., CEA, CA199, c-Met), compared to the control group.

Altogether, these results in PDAC and stomach adenocarcinoma tend to confirm that IL-1RAP plays an important role in the development of solid and hematological cancers.

**Figure 5:**
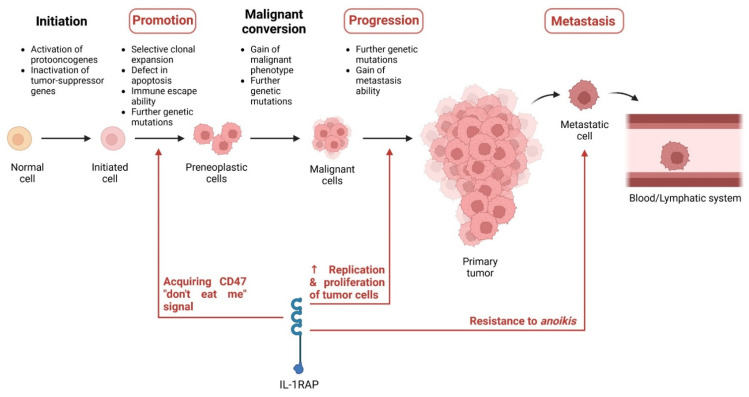
Involvement of IL-1RAP in tumor development. Created with BioRender.com.

These previous experiments studied the impact of IL-1RAP on the general development of tumors. At the same time, other studies explored the role of IL-1RAP in particular phases of tumor development: the immune escape capability, the progression of the tumor, and the *anoikis* resistance for the metastases process (Figure 5).

First, it was observed that IL-1RAP plays an important role during the promotion phase in cervical cancer to acquire immune escape capacity by expressing CD47 (the “don’t eat me” signal) on the surface of tumor cells. Cervical cancers are mainly caused by HPV infection, during which HPV oncoproteins will be produced. The oncoproteins E6 and E7 are the main factors of cervical cancer. They upregulate the splicing factor SRSF10, which in turn will mediate the production of the membrane form of IL-1RAP. As the cervical cancer cells present an overexpression of IL-1β, then the NF-κB pathway will be highly activated by IL-1RAP. It was then demonstrated that this IL-1RAP-mediated NF-κB activation induced the production of CD47 at the surface of tumor cells. CD47 is a glycoprotein that binds to signal regulatory protein alpha on macrophages, thus inhibiting the phagocytosis of cancer cells [112]. Therefore, this study revealed the essential role of IL-1RAP in the development of cervical cancers by promoting the expression of the immune escape signal CD47.

Second, the IL-1RAP role in the progression of tumor cells was observed in vitro in glioma cells (M059 J and U373). This study focused on the impact of protein tyrosine phosphatase receptor-δ (PTPRD) in glioma cells, and it was demonstrated that overexpression of PTPRD induces the upregulation of IL-1RAP. Moreover, under upregulation of PTPRD, an increase in the S and G2 cell cycle phases was observed in glioma cells, demonstrating that PTPRD, possibly through the IL-1RAP pathway, promoted the replication of glioma cells and so the proliferation of the tumor [106]. Furthermore, it is known that IL-1RAP is implicated in synaptic differentiation and neuronal growth [63], which could explain its implication in glioma proliferation. Yet, it must be noted that this study only assessed in vitro the role of PTPRD and IL-1RAP in gliomas. Further in vivo research would be necessary to fully characterize their involvement in this pathology.

Third, IL-1RAP was identified in Ewing sarcoma as a suppressor of *anoikis*. To disseminate and create metastases, tumor cells need to overcome this mechanism, and it was recently demonstrated that IL-1RAP was a key factor of resistance to *anoikis* in Ewing sarcoma. More remarkably, this role seems independent from the IL-1R family pathway (NF-κB). *Anoikis* can be induced by the accumulation of reactive oxygen species (ROS) in the cell as well as oxidized membrane phospholipid (lipid ROS), and a depletion in glutathione (GSH) in the cytoplasm is responsible for ROS and lipid ROS production. Thus, the level of GSH is a key factor in resisting *anoikis*. Two systems exist to maintain this GSH level: (i) retrieving the exogenous cysteine, which is a limiting substrate for GSH synthesis, via the xCT/CD98 transporter (known as X_c_^−^), and (ii) *de novo* synthesis via the transsulfuration (TSS) pathway, which converts serine and methionine to cysteine. In this context, IL-1RAP was found to be involved in both pathways. Firstly, by binding to both xCT and CD98, IL-1RAP will enhance the activation of the X_c_^−^ transporter, resulting in a higher amount of GSH in the cell, thus reducing the production of ROS. Secondly, when exogenous cysteine is depleted, IL-1RAP will activate the cystathionine gamma-lyase (CTH), which is responsible for the *de novo* synthesis of cysteine. The overexpression of IL-1RAP and the activation of one or the other of the pathways lead both in vitro and in vivo to the increase in the local invasion and metastatic capacity of Ewing sarcoma cells. Finally, it was demonstrated that the oncogenic EWS-ETS fusion proteins produced by Ewing sarcoma cells are the effectors of the transcriptional activation of IL-1RAP, resulting in IL-1RAP overexpression [101].

### 3.3. Role in the Tumor Environment

IL-1RAP is involved in the different phases of tumor development by activating signaling pathways in cancer cells. Yet, cancer cells are far from being the only kind of cell that comprise the tumor mass. Indeed, the tumor microenvironment (TME) is composed of multiple different cells and other factors that crosstalk with the tumor cells. This represents many possibilities of interaction between IL-1RAP and the IL-1R family, especially because of their involvement in the inflammation process. 

**Figure 6:**
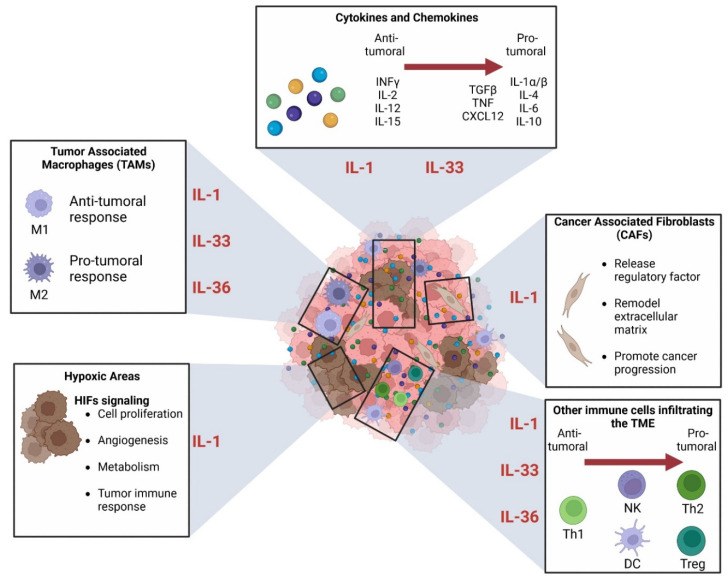
Involvement of the IL-1 family axis in the TME. Created with BioRender.com.

The inflammation is now considered a hallmark of cancer, and its impact on tumor development is driven by many interconnected factors that are found in the TME (Figure 6): cytokines, chemokines, innate and adaptive immune cells, fibroblasts, and hypoxic areas. The TME was described to represent the majority of the tumor mass, and its involvement in cancer development is complex. The TME will grow alongside the tumor cells, and they both impact each other’s development [121].

The cytokines are a group of many small proteins that are involved in cell signaling and autoimmune response. Cytokines include prostaglandins, chemokines, interleukins (IL), interferons (IFN), lymphokines, transforming growth factors (TGF) and tumor necrosis factors (TNF). They can be produced by many cells, including fibroblast, stromal cells and immune cells, which can be found in high amounts in the TME. Some cytokines will present a pro-inflammatory (anti-tumoral) action, while others can present an immunosuppressive (pro-tumoral) function. The balance between pro- and anti-tumoral activities within the TME will depend on the stage of development of the tumor [17]. 

The chemokines may have several roles in the growth of the TME. They are mostly described as key factors for the recruitment and migration of the leucocytes to the inflammatory sites. Recently, it was described that chemokine can not only recruit leucocytes but might also be involved in the polarization of CD4+ T cells [122].

Another important component of the TME is the immune cells that are recruited in the early stages of tumor development. Both innate and adaptive immune systems are involved in TME homeostasis, mainly by secreting anti-inflammatory cytokines. For example, macrophages are well-known to be involved in tumor progression and metastasis: tumor-associated macrophages (TAMs) can be split into two activated phenotypes, (i) the anti-tumoral M1 (classical activation), which causes cytotoxicity or antibody-dependent cell-mediated cytotoxicity (ADCC), and (ii) the M2 (alternative activation) which promote the tumor progression and metastasis by inhibiting T cell-mediated immune response and stimulating tumor angiogenesis [123,124]. 

The most abundant cells in the TME are cancer-associated fibroblasts (CAFs). CAFs affect tumor initiation and development thanks to various mechanisms. They are involved in the regulation of tumor and stromal cell biology via cell-cell contact, release several regulatory factors, and can synthesize and remodel the extracellular matrix. Thus, CAFs are critically involved in cancer progression [125,126]. Furthermore, it was observed that CAF phenotypes could present two subtypes: (i) “inflammatory CAFs” (iCAFs) [127] that show IL-6 and leukemia inhibitory factor (LIF) markers, and (ii) “myofibroblastic CAFs” (myCAFs) with alpha-smooth muscle actin (αSMA) marker. MyCAFs are found adjacent to tumor cells, while iCAFs are found farther away. For example, the presence of both subtypes was observed in human PDAC [128].

Finally, another important component of the TME is the presence of hypoxic areas. Hypoxia is known to be a feature of aggressiveness in solid and hematological cancers as it will impact cell proliferation, angiogenesis, metabolism, and tumor immune response. These effects are mainly driven by hypoxia-inducible factor (HIF) signaling, which acts to maintain an immunosuppressive environment within the TME [121,129,130,131].

All these components of the TME act mostly as pro-tumoral factors and are key factors in determining the aggressiveness of tumors. TME components can therefore be used as new targets for innovative therapies. One example, among many others, is the use of hypoxic areas to activate prodrugs to treat solid or hematological cancers, as discussed in a review by Baran and Konopleva [132].

Unfortunately, no study has yet assessed the direct impact of IL-1RAP on the different components of the TME. Nevertheless, it was shown that IL-1RAP is essential for IL-1R [133] and the IL-33R [134] signaling pathways. It was also demonstrated that a single nucleotide substitution (A471T) of IL-36R downregulates the IL-36 signaling by decreasing the interaction between IL-36R and IL-1RAP [61]. Furthermore, it has been described that expression levels of type II IL-1 receptor (IL-1R II), which works as a decoy receptor for IL-1, were found to decrease in cervical cancers [135]. The IL-1 superfamily, through IL-1/33/36 signal, has been shown to play a crucial role in the development of several types of cancer, most probably through the inflammatory processes, it mediates in the TME. 

As IL-1RAP involvement in the TME has not yet been assessed but showed an essential role in IL-1/33/36 signal transduction, we will now focus on the implication of these three pathways in the TME components.

#### 3.3.1. Connection between IL-1 Superfamily and Immune Cells in TME

The IL-1R axis has been shown to play an important role in several immune cells in the TME (Figure 6). 

Firstly, it was observed in melanoma that crosstalk between tumor cells, macrophages, and fibroblasts of the TME would initiate an IL-1 signaling cascade, which generates a CXCR2-stimulating secretome. Ultimately, this will lead to enhanced melanoma cell survival against MAPK signaling inhibition therapy (e.g., vemurafenib, selumetinib) [136].

Secondly, the IL-1β/IL-1R pathway is known to be essential for the early differentiation and the proliferation of Th17 cells. Moreover, these Th17 cells will produce IL-17, which promotes tumor growth and angiogenesis [137] in murine melanoma and bladder carcinoma.

Thirdly, in HPV-negative oropharyngeal squamous cell carcinoma (OPSCC), it has been demonstrated that the IL-1/IL-1R axis is responsible for the production of the chemokine CXCL8, which will recruit neutrophil cells to the TME. These tumor-associated neutrophil cells (TAN) are associated with poor prognosis in OPSCC [138].

The IL-33R axis also presented a great role in tumorigenesis, as described in a review from Fournié and Poupot [139].

In addition to its direct effect on the tumor cells, promoting their proliferation and tumor growth, IL-33 is involved in the TME to promote angiogenesis, matrix remodeling, and cytokines and growth factors produced by the TME components.

First, IL-33 stimulates mast cells to initiate a pro-inflammatory response by secreting cytokines and leukotrienes, which can lead to the initiation of the tumorigenesis process. Furthermore, IL-33 induces the accumulation of mast cells in tumors. Second, the IL-33 signaling on the infiltrating immune cells induces the production of IL-6, a pro-tumorous cytokine. Third, IL-33 is responsible for the recruitment of macrophages and also induces their polarization into M2 phenotype TAMs. Then, the IL-33-stimulated TAMs promote the intravasation process of the tumor cells. Finally, IL-33 signaling presents a direct effect on regulatory T cells (Treg) by enhancing TGF-β1-mediated differentiation. Thus, IL-33 may provide a signal to induce Treg accumulation and maintenance in inflamed tissues. However, depending on the context, this accumulation of Treg can either present an antitumor effect or a pro-tumorigenic effect. Besides, IL-33 signaling may present a direct antitumor function as well. Indeed, the inhibition of proliferation and induction of apoptosis was observed in vitro in human pancreatic cancer cell line MIA PaCa-2 under treatment with IL-33.

Regarding the IL-36R axis, it also presented both anti- and pro-tumorigenic functions.

Firstly, in an inflammatory context, it has been demonstrated that IL-36β will mainly stimulate M2 macrophages and not M1 macrophages. Moreover, higher expressions of IL-36R mRNA were found in M2 than in M1 macrophages. Finally, under IL-36β stimulation, M2 macrophages produce pro-inflammatory cytokines (e.g., IL-1β, IL-8, TNF-α), although in vitro-activated M2 macrophages also produce IL-1Ra, which impairs responsiveness to IL-1β [140]. Secondly, several studies assessed the role of the IL-38 or IL-36/IL-36R axis in colorectal cancer (CRC). Both IL-36α and IL-36γ have been described with anti-tumorigenic function [141,142]. Indeed, in the TME, IL-36γ is produced by M1 macrophages [141] and promotes the memory T cells response, which is associated with a positive prognosis in CRC [143]. Yet, it can be noticed that high expression of IL-36γ was also described as being associated with a low survival rate in CRC [141]. Then, the IL-36R antagonist (IL-36Ra) expression was associated with poor prognosis and lower intratumoral B cells and memory T cells response [143]. Finally, the IL-38 axis presents opposite effects depending on the type of cancer. Indeed, it was found to decrease and be associated with anti-tumor function in CRC, while in PDL-1-negative lung carcinoma, IL-38 was associated with a pro-tumorigenic effect [141,144].

#### 3.3.2. Role in Hypoxia

Only one study in TNBC assessed the relationships between hypoxia and the IL-1 axis in TME [145]. It has been demonstrated that hypoxia plays a major role in tumor invasiveness and metastasis process by promoting the HIF-1α/GPER (G protein estrogen receptor) and IL-1β/IL-1R axes.

Under hypoxia, HIF-1α production will increase, leading to the upregulation of GPER. Then, cooperation between HIF-1α and GPER will promote the expression of IL-1β, which in return will activate the IL-1R signaling pathway. This activation will increase the expression of metastatic genes and invasive properties of the TNBC cells, thus explaining the correlation between TNBC aggressiveness and the overexpression of both HIF-1α and IL-1β in patient samples that showed poor prognosis.

Moreover, it was demonstrated that the activation of the IL-1/IL-1R axis under hypoxia also promotes an invasive CAFs phenotype within TNBC-associated TME [145].

#### 3.3.3. The Relationship between IL-1 Superfamily and the Cancer-Associated Fibroblasts (CAFs)

As already mentioned, CAFs can be sorted into two phenotypes, iCAFs (away from the tumor cells) and myCAFs (found next to tumor cells).

The impact of the IL-1R signaling pathway on CAFs has been recently explored in PDAC.

Firstly, it was demonstrated that IL-1 induces JAK/STAT signaling in CAFs, which promotes the formation of the iCAF phenotype. Yet, tumor cells produce TGF-β, which downregulates the expression of IL-1R1 on local CAFs. Under the downregulation of IL-1R1, JAK/STAT signaling will decrease in CAFs, which promotes their myCAFs phenotype. Thus, tumor cells induce a local regulation that explains the localization of iCAFs and myCAFs in the TME [146].

Secondly, tumor cells will also produce IL-1α, which will stimulate CAFs to keep an inflammatory state within the TME by promoting their production of inflammatory factors. IL-1α will especially induce the production of CXC chemokines by CAFs. This was demonstrated in vitro as the inhibition of IL-1α led to a decrease in the expression of CXC chemokine in PDAC cells and CAFs [147].

Altogether, these results demonstrate the major and complex involvement of the IL-1 superfamily in the TME as the different axis can play both anti- or pro-tumoral functions, depending on the type and grade of cancer, the state of inflammation, and the cells with which they interact (Figure 6). Now, the impact of IL-1RAP was not explored in these studies, although it is essential to promote the signaling pathway of the IL-1 family. Further research in this field could show great potential to elucidate pathogenesis mechanisms or to identify new therapeutic targets.

## 4. Therapies Targeting IL-1RAP

As IL-1RAP is found to be overexpressed in multiple cancers and seems to be involved in the physiopathology of other diseases as well. Several teams worked on IL-1RAP as a new target for therapies. For example, the impact of the blockade of IL-1RAP on the IL-1/33/36 signaling was recently assessed to investigate the potential of IL-1RAP antibody-based therapy in inflammatory diseases [113]. This study showed that the production of pro-inflammatory cytokines (such as IFN-γ and IL-6) is more inhibited by the blockade of IL-1RAP than by the blockade of the primary receptor alone (IL-1R, ST2, IL-1Rrp2). This suggests that IL-1RAP could be an effective target for therapies in diseases influenced by several IL-1 family members.

Another important element to consider about IL-1RAP and the IL-1 family is their involvement in anti-cancer therapies escape. Indeed, the expression of IL-1RAP has been described as a possible biomarker to predict the response to tyrosine-kinase inhibitors (TKI) therapy in CML. Indeed, patients with low levels of IL-1RAP expression were found to be more likely to respond positively to TKI therapy after 6 months of treatment [148]. Moreover, it was recently observed in vivo that the production of IL-1β by M0 macrophages reduces the sensitivity to neoadjuvant chemotherapy in osteosarcoma [149].

In this context, various kinds of therapies are now in preclinical and clinical development. Most of them are based on blocking antibodies targeting IL-1RAP. The others include chimeric antigen receptor T cells (CAR-T) therapy and the development of an antibody with theranostic ability. Table 3 and Table 4 summarize these preclinical and clinical studies.

### 4.1. Anti-IL-1RAP Antibody in Cancers, Alone and in Combination

A Chinese team recently worked on an antibody fragment (scFv 12H7) targeting IL-1RAP in a preclinical model of TBNC. Zheng et al. showed that scFv 12H7 could inhibit the development of TNBC-derived tumor cells both in vitro and in vivo. They concluded that the scFv 12H7 could represent a potential therapeutic candidate but still need further investigations [24].

A Swedish company Cantargia AB, located in Lund, worked for several years on IL-1RAP as a target in various cancers. They investigated IL-1RAP in chronic and acute myeloblastic leukemias (CML & AML) and also in several solid tumors. They are now clinically testing the efficacy of ADCC and the blockade of IL-1RAP induced by anti-IL-1RAP antibodies in those pathologies. These clinical trials are described in the next paragraphs of this review, and the results of these studies are highly anticipated. Because IL-1RAP is highly involved in the inflammatory process, particular attention should be paid to the safety and side effects of these treatments, such as autoimmune diseases. Cantargia AB published interim safety results for their clinical trial CANFOUR (NCT03267316) and demonstrated that their antibody, Nadunolimab (alternative names Nidanilimab and CAN04), is well-tolerated as no acute toxicity was reported [26,150,151].

**Table 3:** Therapies targeting IL-1RAP that underwent preclinical studies.

Anti-IL-1RAP Treatment	Study	Pathology	Model	Outcomes	References
Fragment of antibody (scFv 12H7)	In vitro & in vivo (on mice)	TNBC	MDA-MB-231 HCC-70	Inhibition of the development of TNBC-derived tumors both in vitro and in vivo	[24]
Full antibody	In vitro & in vivo (on mice)	CML	KU812 (in vitro) KG-1 (in vitro) CML patients’ cells (in vitro & in vivo) BV-173 (in vivo)	Inhibition of IL-1 signaling and expansion of primitive CML cells	[93,152]
Full antibody (mAb81.2)	In vitro	AML	AML patient cells	The antibody can induce ADCC But does not block IL-1 signaling	[96]
Full Antibodies (mAb81.2 & mAb3F8)	In vitro & in vivo (on mice)	AML	MA9RasAML patient cells	Antibodies can induce ADCC mAb3F8 can block IL-1 signaling (in vitro)	[153]
Full antibody (Nadunolimab) with chemotherapy	In vitro & in vivo (on mice)	NSCLC	LU2503 (PDX)	Nadunolimab works in synergy with chemotherapy by blocking the IL-1α and IL-1β signaling pathway	[28]
CAR-T cells (IL1RAP CAR-T)	In vitro & in vivo (on mice)	CML	KU812 cells K562 cells	Cytotoxic activity of autologous CAR-T against CML cells	[27]
	In vitro & in vivo (on mice)	AML	HL-60 cells Molm-13 cells MonoMac-6 cells PDX	Cytotoxic activity of autologous CAR-T against AML cells	[154]

**Abbreviations:** ADCC: antibody-dependent cell-mediated cytotoxicity; AML: acute myeloid leukemia; CAR: chimeric antigen receptor; CML: chronic myeloblastic leukemia; NSCLC: non-small cell lung cancer; PDX: patient-derived xenograft; TNBC: triple-negative breast cancer.

**Table 4:** Therapies targeting IL-1RAP that underwent clinical trials.

Anti-IL-1RAP Treatment	Treatment in Combination	Clinical Phase	Pathology	CURRENT STATUS	ClinicalTrial ID (Study Name)
Full antibody (Nadunolimab)	Cisplatin Gemcitabine Nab-paclitaxel	I/IIa	PDAC (phase I/IIa) NSCLC (phase I/IIa) TNBC (phase I) CRC (phase I)	Phase I: Completed Phase IIa: Active, not recruiting (PDAC) Recruiting (NSCLC)	NCT03267316 (CANFOUR) [26,150,151]
Full antibody (Nadunolimab)	Pembrolizumab	I	NSCLC UC HNSCC Malignant Melanoma	Active, not recruiting	NCT04452214 (CIRIFOUR)
Full antibody (Nadunolimab)	FOLFIRINOX	I	Metastatic PDAC	Active, not recruiting	NCT04990037 (CAPAFOUR)
Full antibody (Nadunolimab)	mFOLFOX DTX G/C	I/II	Advanced solid tumors CRC NSCLC BTC	Active, not recruiting	NCT05116891 (CESTAFOUR)
Full antibody (Nadunolimab)	Carboplatin Gemcitabine	I/II	TNBC	Recruiting	NCT05181462 (TRIFOUR)
CAR-T cells	None	N/A	CML	Completed	NCT02842320 (CAR-LMC)
CAR-T cells	None	N/A	AML	Recruiting	NCT04169022 (CAR-LAM)

**Abbreviations:** AML: acute myeloid leukemia; BTC: biliary tract cancer; CAR: chimeric antigen receptor; CML: chronic myeloblastic leukemia; CRC: colorectal cancer; DTX: docetaxel; FOLFIRINOX: folinic acid, fluorouracil, irinotecan, oxaliplatin; G/C: gemcitabine/cisplatin; HNSCC: head and neck squamous cell carcinoma; ID: identifier; mFOLFOX: modified FOLFOX (Oxaliplatin, 5-fluorouracil, leucovorin); N/A: not applicable; NSCLC: non-small cell lung cancer; PDAC: pancreatic ductal adenocarcinoma; TNBC: triple-negative breast cancer; UC: urothelial carcinoma.

i. Preclinical in vitro and in vivo study in CML

In their studies, Järås et al. [93] & Ågerstam et al. [152] have identified IL-1RAP as a unique cell surface biomarker that is able to distinguish candidate CML stem cells from normal hematopoietic stem cells (HSC). Indeed, they showed that HSCs do not express IL-1RAP, which would avoid any severe negative effects on normal hematopoiesis when using therapies that target IL-1RAP. They tested a polyclonal rabbit anti-human IL-1RAP antibody in CML to induce antibody-dependent cell-mediated cytotoxicity (ADCC) of the CML cells. Using IL-1RAP as a target, they were able to preferentially focus ADCC on the CML cells that presented the Philadelphia mutation. Another advantage of this therapy is its independence regarding the tyrosine-kinase inhibitors (TKIs) resistance, as it could be used to kill the cells that acquired TKIs resistance. Finally, their results in vivo showed that the antibody was still able to target primitive CML cells even if the patient were under TKI treatment. Hence, a bimodal therapy that combines TKIs with an anti-IL-1RAP antibody might improve therapeutic options for refracting CML patients.

ii. Preclinical in vitro and in vivo study in AML

Askmyr et al. [96] explored the involvement of IL-1RAP in AML. Firstly, they demonstrated that both immature and mature AML cells present an up-regulated expression of IL-1RAP, which could become a new therapeutic target in this disease. Secondly, they tested in vitro the efficacy of anti-IL-1RAP antibodies (mAb81.2). They showed their antibody could induce ADCC against AML cells, yet it did not seem to block the IL-1 signaling.

This team produced a new antibody (mAb3F8) able to block IL-1 signaling, and Ågerstam et al. [153] tested its efficacy in vivo in xenograft models of AML. The antibody significantly reduced the leukemic burden. Yet, only ADCC action could be assessed in vivo for two major reasons: (i) human cells are known to respond poorly to murine IL-1 and (ii) the grafted cells (MA9Ras) did not respond to IL-1 stimulation. Hence, in vitro experiments were carried out to explore the IL-1 blockade by mAb3F8 on AML cell proliferation. They demonstrated that the antibody significantly suppressed the proliferation of primary AML cells. Finally, even if IL-1RAP expression was weak or absent in the majority of normal human tissues, further in vivo toxicity studies are needed to establish a proper safety profile of the antibody.

iii. The CAN04 antibody (Nadunolimab) in the clinical trials of Cantargia AB

Based on their preclinical results, Cantargia AB developed a fully humanized and ADCC-enhanced monoclonal antibody that targets IL-1RAP called CAN04 [155]. It shows two mechanisms of action: (i) by directly blocking IL-1RAP and, therefore, its intracellular signals, and (ii) through ADCC against IL-1RAP. Cantargia AB started five clinical trials between 2017 and 2022 in order to assess the safety and efficacy of CAN04 (later named Nadunolimab) in several solid cancers:

1. Started in August 2017, the CANFOUR clinical trial (ClinicalTrial ID NCT03267316) is a phase I/IIa open study. It is conducted in several European countries (28 locations among 11 countries). The goal is to assess the safety and efficacy of CAN04 in solid tumors. The study is separated into two parts: (i) to assess the safety and tolerability of the product and to evaluate the maximum tolerated dose and recommended phase 2 dose (MTD/RP2D) in PDAC, NSCLC, TNBC, and CRC [26]; and (ii) to evaluate the safety and tolerability at RP2D level in an expanded cohort in PDAC and NSCLC patients. This study aims to enroll 140 participants;
2. Starting in September 2020, this second clinical trial on CAN04 (ClinicalTrial ID NCT04452214) is a phase I open-label study. The goal is to assess the safety and to establish a recommended dose of CAN04 in combination with pembrolizumab for the treatment of incurable or metastatic NSCLC, head and neck squamous cell carcinoma, urothelial cancer, or malignant melanoma. This study expects to enroll 15 participants;
3. Started in July 2021, the CAPAFOUR study (ClinicalTrial ID NCT04990037) is a phase I open-label study. This clinical trial aims to determine the safety and effectiveness of CAN04 in combination with a modified FOLFIRINOX protocol in metastatic PDAC. The study expects 50 participants;
4. The fourth study started in September 2021 (ClinicalTrial ID NCT05116891), and it is a phase I/II open-label study. The phase I goal is to assess the safety, tolerability and MTD/RP2D of CAN04 in combination with standard chemotherapies (mFOLFOX, gemcitabine/cisplatin, docetaxel), while phase II will aim to assess the preliminary efficacy of CAN04 in combination with the same chemotherapies. The two phases will focus on locally advanced or metastatic CRC, NSCLC, or biliary tract cancer. This study aims to enroll 180 participants;
5. The last study, TRIFOUR, started in January 2022 (ClinicalTrial ID NCT05181462) and is a phase I/II randomized open-label study. The phase I goal is to assess the safety and highest dose without the serious side effects of CAN04 in combination with gemcitabine and carboplatin. Phase II will aim to assess the efficacy of CAN04 in combination with the same chemotherapy. The two phases will focus on patients with advanced or metastatic TNBC. This study expects to enroll 18 patients in phase I and 98 patients in phase II.

### 4.2. Anti-IL-1RAP Chimeric Antigen Receptor T (CAR-T) Cells in Cancers

As already illustrated by the team of Cantargia AB, IL-1RAP is an interesting target in both AML and CML [93,95,96,97,152,153]. In order to explore another option to treat these diseases, a team from Besançon, France, developed CAR-T cells able to target IL-1RAP.

Using cellular engineering, CAR-T cells combine the specificity of mAbs with the killing ability of T lymphocytes. CAR-T cells are born from the combination of CD8+ T lymphocytes (killer T cells) or CD4+ T lymphocytes (helper T cells) and the antigen receptor of antibodies. After its engineering, the CAR gene is generally introduced within the T cells genome by plasmid transfection. Then, the T cells will synthesize and express CARs at their membrane, allowing them to specifically target tumor cells. Once the CAR-T recognizes its target, the T cell is activated via intracellular signal transduction. Then, multiple killing mechanisms are involved [156]: degranulation of perforin and granzymes, Fas and Fas-Ligand pathway, and cytokine production. Due to their cytotoxic properties, CAR-T cells may exert undesired toxicities. The three most important side effects of CAR-T therapy are [157,158]: cytokine release syndrome (CRS), neurotoxicity, and on-target/off-tumor recognition. About the latter, IL-1RAP shows its full potential as it is a rare cell-surface protein that is over-expressed in CML and AML cells [19,159].

#### 4.2.1. Preclinical Studies

Warda et al. [27] first worked in CML models to design and test their anti-IL-1RAP CAR-T cells (IL1RAP CAR-T). Firstly, they demonstrated in vitro that their CAR-T was able to specifically target the primary CML cells associated with an activation of the T cell leading to cell death with 95% efficiency (effector: target ratio of 3:1) compared to an alloreactive cytotoxicity of 18% and 21% for CFSE-stained cells and Mock-T cells (T cells that went under the same genetic modification that the CAR-T cells, but without the CAR gene) respectively. Secondly, they assessed the efficacy of IL1RAP CAR-T in vivo in two CML models (xenograft of KU812 or K562 cells) in comparison to untreated and Mock-T-treated mice (mice treated with Mock-T cells instead of CAR-T cells). All the CAR-T-treated mice presented a complete elimination of the tumor with no death recorded, whereas the untreated and Mock-T-treated mice showed tumor progression leading to death for 2/3 mice at D28. Simultaneously, the team engrafted NOG mice with human cord blood CD34+ cells to assess the impact of IL1RAP CAR-T on healthy hematopoietic stem cells (HSCs). The results showed that HSCs were not affected by IL1RAP CAR-T, which indicates that this therapy should display few side effects on the hematopoietic system. 

Then, the same team also worked on AML models, based on the literature which has shown that IL-1RAP is over-expressed in this pathology [95,96,97] and that anti-IL-1RAP antibody therapy proved its efficacy in preclinical studies [97,153]. First, they demonstrated that IL-1RAP could be expressed at different levels on AML cell lines and primary AML cells [154]. Thus, they selected three cell lines with low, intermediate, or high IL-1RAP expression, respectively HL-60, Molm-13 and MonoMac-6 cells, to test their CAR-T therapy on cells with different levels of expression. In vitro, IL-1RAP CAR-T cells were able to eliminate more than 95% of the AML cells, independently from their level of IL-1RAP expression, in comparison to a positive control (CML cells KU812, 99%) and a negative control (IL-1RAP-negative K562 cells, 10,5%). Then, they tested their CAR-T in vivo against xenografts of the three AML cell lines in NSG mice and PDX cells in NSGS mice. For each experiment, they compared the CAR-T efficacy against untreated mice and C0T-treated mice (C0T are untransduced-T cells). For AML cell lines, CAR-T cells were able to reduce the AML cell growth (partially for Molm-13, which presents intermediate IL-1RAP expression). In PDX models, CAR-T therapy significantly increased the overall survival of mice (240 days) compared to untreated (98 days) and C0T-cell-treated mice (126 days) [154].

#### 4.2.2. Clinical Trials

In keeping with these results, the French team was allowed to start two clinical trials:

1. The CAR-LMC clinical trial (ClinicalTrials ID NCT02842320): started in October 2015, this trial enrolled 53 patients and monitored them for 24 months in an open-label single-armed study. The first step was to collect T-cells from the patient and engineer them with a CAR. Then, they infused them back into the patient and started the monitoring with blood samples. These samples were analyzed by flow cytometry to detect IL-1RAP expression on the surface of cells. At the time of writing this review, the study is ending, and no results have been published yet;
2. The CAR-LAM clinical trial (ClinicalTrials ID NCT04169022): started in July 2019, this trial is still recruiting, and the team aims for the enrollment of 50 patients. It is an open-label, non-randomized two-armed study. The first arm is comprised of AML patients at diagnosis (except AML3), while the second arm is made up of AML patients at relapse after chemotherapy, targeted therapy, or allografts. The protocol will be similar to the CAR-LMC study: a two-year follow-up with flow cytometry analysis to explore the IL-1RAP expression of cells in blood samples. At the time of writing this review, the study is starting, and no results have been published yet.

## 5. Conclusions

In the last decade, new findings highlighted the role of IL-1RAP within the IL-1 superfamily signaling pathway and its role in tumor development and progression. IL-1RAP is now considered a promising therapeutic target in several hematological and solid cancers. Moreover, IL-1RAP-specific therapies were recently developed: two antibody-based therapies and one CAR-T cell therapy. Nevertheless, further studies are needed to better characterize the effects of IL-1RAP blockade in different types of cancers. Since IL-1RAP is also shown to be involved in the pathogenesis of inflammatory diseases, the blockade of IL-1RAP might also be a strategy to treat those non-cancer disorders. Alternative strategies also remain to be explored, such as (i) the development of small molecules that could antagonize IL-1RAP ecto- or endo-domains; (ii) antibody-drug conjugates (ADC) that combine the action and targeting properties of an antibody with the cytotoxic action of chemotherapy; and (iii) the development of theranostic agents, which could be labeled with various radionuclides to achieve diagnostic and/or therapeutic purposes. For the latter strategy, our team recently started the development of such a compound and has already assessed its diagnostic properties in a preclinical model of AML [160]. 

## Figures and Tables

**Table 1:** Cytokines member of the interleukin 1 (IL-1) superfamily.

Cytokine	FAMILY NAME	Known Structure (Uniprot ID *)	Biological Effect	Reference
IL-1α	IL-1F1	Yes (P01583)	Proinflammatory	[21,29,30,31]
IL-1β	IL-1F2	Yes (P01584)	Proinflammatory	[21,29,31,32]
IL-1Ra (Anakinra)	IL-1F3	Yes (P18510)	Anti-inflammatory	[21,29,33]
IL-18	IL-1F4	Yes (Q14116)	Proinflammatory	[21,34,35,36]
IL-33 (NF-HEV *)	IL-1F11	Yes (O95760)	Proinflammatory	[21,34,37,38]
IL-36α	IL-1F6	Yes (Q9UHA7)	Proinflammatory	[21,39,40]
IL-36β (IL1-H2)	IL-1F8	No (Q9NZH7)	Proinflammatory	[21,39]
IL-36γ	IL-1F9	Yes (Q9NZH8)	Proinflammatory	[21,39,41]
IL-36Ra	IL-1F5	Yes (Q9UBH0)	Anti-inflammatory	[21,39,42]
IL-37	IL-1F7	Yes (Q9NZH6)	Anti-inflammatory	[21,43,44]
IL-38	IL-1F10	Yes (Q8WWZ1)	Anti-inflammatory	[21,45,46,47]

* ID: identifier; NF-HEV: nuclear factor from high endothelial venules. Table adapted from Fields et al. [21].

**Table 2:** Receptors and co-receptors of the interleukin 1 (IL-1) superfamily.

Receptor (RNA Tissues Expression) *	Co-Receptor (RNA Tissues Expression) *	Signaling Pathways	Ligand	Reference
IL-1RI (ubiquitous)	IL-1RAP ** (ubiquitous)	MyD88 **: MAPK ** (MAP3K **) NF-κB **, AP-1 **	IL-1α IL-1β IL-1Ra	[20,48,49]
IL-1RII (appendix, bone marrow, colon, esophagus, placenta, skin, stomach, spleen)	IL-1RAP (ubiquitous)	MyD88: MAPK NF-κB, AP-1	IL-1α IL-1β IL-1Ra	[20,48,49]
IL-18Rα (ubiquitous)	IL-18RAP (bone marrow, appendix, spleen)	MyD88: MAPK (JNK1 **) NF-κB, AP-1	IL-18	[20,35,48,49]
	SIGIRR ** (ubiquitous, spleen, kidney)	STAT3**: MAPK, NF-κB, AP-1 (inhibition)	IL-37	[20,35,48,49]
IL-33R (placenta, kidney, lung, adrenal, gall bladder)	IL-1RAP (ubiquitous)	MyD88: MAPK, NF-κB, AP-1	IL-33	[20,48,49]
IL-36R (ubiquitous, skin, thyroid, esophagus, kidney)	IL-1RAP (ubiquitous)	MyD88: MAPK, NF-κB, AP-1	IL-36α IL-36β IL-36γ IL-36Ra IL-38	[20,48,49,50]
Unknown	IL-1RAPL1 ** (brain)	PSD-95 **, JNK **	Unknown	[20,48,49,51,52]
Unknown	IL-1RAPL2 (adrenal, urinary bladder, testis, brain)	Unknown	Unknown	[20,48,49,53]

* RNA expressions from the BioProject “HPA RNA-seq normal tissues” (BioProject ID: PRJEB4337). ** AP-1: activator protein 1; IL-1RAP: interleukin 1 receptor accessory protein; IL-1RAPL: interleukin 1 receptor accessory protein-like; JNK: c-Jun N-terminal kinase; MAP3K: mitogen-activated protein kinase kinase kinase; MAPK: mitogen-activated protein kinase; MyD88: myeloid differentiation primary response 88; NF-κB: Nuclear factor kappa-light-chain-enhancer of activated B cells; PSD-95: postsynaptic density protein 95; SIGIRR: Single Ig IL-1-related receptor; STAT3: Signal transducer and activator of transcription 3. Table adapted from Fields et al. [21].

## Data Availability

Not applicable.
